# Intraoperative radiotherapy: review of techniques and results

**DOI:** 10.3332/ecancer.2017.750

**Published:** 2017-06-29

**Authors:** Avinash Pilar, Meetakshi Gupta, Sarbani Ghosh Laskar, Siddhartha Laskar

**Affiliations:** Department of Radiation Oncology, Tata Memorial Hospital, Dr Ernest Borges’ Marg, Parel, Mumbai, MS, India 400012

**Keywords:** IORT, techniques, indications, outcomes, complications

## Abstract

Intraoperative radiotherapy (IORT) is a technique that involves precise delivery of a large dose of ionising radiation to the tumour or tumour bed during surgery. Direct visualisation of the tumour bed and ability to space out the normal tissues from the tumour bed allows maximisation of the dose to the tumour while minimising the dose to normal tissues. This results in an improved therapeutic ratio with IORT. Although it was introduced in the 1960s, it has seen a resurgence of popularity with the introduction of self-shielding mobile linear accelerators and low-kV IORT devices, which by eliminating the logistical issues of transport of the patient during surgery for radiotherapy or building a shielded operating room, has enabled its wider use in the community.

Electrons, low-kV X-rays and HDR brachytherapy are all different methods of IORT in current clinical use. Each method has its own unique set of advantages and disadvantages, its own set of indications where one may be better suited than the other, and each requires a specific kind of expertise.

IORT has demonstrated its efficacy in a wide variety of intra-abdominal tumours, recurrent colorectal cancers, recurrent gynaecological cancers, and soft-tissue tumours. Recently, it has emerged as an attractive treatment option for selected, early-stage breast cancer, owing to the ability to complete the entire course of radiotherapy during surgery. IORT has been used in a multitude of roles across these sites, for dose escalation (retroperitoneal sarcoma), EBRT dose de-escalation (paediatric tumours), as sole radiation modality (early breast cancers) and as a re-irradiation modality (recurrent rectal and gynaecological cancers).

This article aims to provide a review of the rationale, techniques, and outcomes for IORT across different sites relevant to current clinical practice.

## Background

Intraoperative radiation therapy (IORT) constitutes delivery of radiation to the tumour/tumour bed while the area is exposed during surgery. IORT is capable of delivering high doses of radiation, precisely to the tumour bed with minimal exposure to the surrounding healthy tissues.

Abe *et al.* from the University of Kyoto, Japan, were the first to introduce IORT in the early 1960s reporting its use in various intra-abdominal tumours [1–3].

IORT is typically used in combination with other modalities like maximal surgical resection, external beam radiotherapy (EBRT) or chemotherapy as a part of the multidisciplinary approach.

Efficacy of IORT has been reported in a wide variety of sites like locally advanced and recurrent rectal cancer, retroperitoneal sarcoma, pancreatic cancer, early breast cancer, and selected gynaecologic and genitourinary malignancies.

## Rationale for the use of IORT

Traditionally, surgery is followed by EBRT in most solid tumours for the elimination of any microscopic residual disease and reducing the risk of local recurrence. However, EBRT in the post-operative setting has the following drawbacks:

- The usual delay between the surgical removal of the tumour and EBRT may allow repopulation of the tumour cells.
- Difficulty in tumour bed localisation or use of larger margins, which may increase normal tissue morbidity.

Most solid tumours exhibit a dose–response relationship, the likelihood of local control improving with increasing dose; however, there are limitations to the doses that can be delivered even with conformal EBRT techniques due to the presence of dose-limiting structures adjacent to the tumour/tumour bed. Especially, in the setting of gross residual disease, doses with EBRT may never be sufficient to achieve adequate local control without causing significant morbidity.

### IORT allows

- Precise localisation of the tumour bed and targeted delivery of high-dose radiation to the tumour bed.
- Minimal exposure of the dose-limiting normal tissues that are displaced away from the tumour bed and shielded from radiation.
- Opportunities for dose escalation beyond that which can be achieved with EBRT.
- Opportunities for re-irradiation especially in recurrent cancers where further irradiation with EBRT may not be possible.

Thus, IORT can deliver higher total effective dose to the tumour bed, facilitate dose escalation without significantly increasing normal tissue complications and improve therapeutic ratio compared with EBRT.

IORT may be used alone or in combination with conventionally fractionated EBRT. Most centres use it in combination with EBRT, as it seems to provide the best therapeutic ratio (decreased risk of late normal tissue damage due to the use of fractionation for some part of the dose).

## Methods of IORT

Several methods have been used to deliver IORT. Electron beams (electron IORT/IOERT), X-rays (kV IORT) and High-dose-rate brachytherapy (HDR IORT) are some of the commonly used methods for the delivery of IORT in current clinical practice.

### Electron IORT

Introduction of electron IORT (IOERT) marked the beginning of the IORT era in the early 1960s [3, 4]. Using variable electron energies depth dose distribution could be controlled to provide uniform dose to target area. However, patients needed to be transported from the operating room (O.R) to the radiation department during surgery, posing logistical issues related to transportation and sterilisation [2, 5]. These problems were overcome with the use of dedicated IOERT facilities, which were quite expensive because of added costs of shielding the O.R and dedicated linear accelerator requirements, limiting their use to few centres in the United States and Europe. The advent of miniaturised, self-shielded, mobile linear accelerators [6] (Novac7, Hitesys SPA, Aprillia, Italy; 7–10 MeV and the Mobetron, IntraOp Medical Corporation, Sunnyvale, CA, USA; 4–12 MeV) in the 1990s, has brought about resurgence of IORT and allowed its use in many centres across the world while reducing the costs. Greater depth of penetration and dose homogeneity relative to HDR-IORT or kV IORT is possible with these devices. They come with applicators of different shapes and sizes, for the treatment of various sites and can deliver the treatment in a matter of minutes [7]. However, these applicators are rigid, thus challenging to use in difficult sites (pelvis and narrow cavities) and can treat a maximum diameter of 15 cm only, larger volumes requiring multiple, closely placed fields. Abutment of fields to treat a wider area is made possible by the use of rectangular applicators or D-shaped applicators called ‘Squircle’.

### HDR IORT

HDR brachytherapy offers distinct dosimetric advantages due to its steep dose fall off and has the ability to deliver high doses to the tumour bed while reducing doses to nearby critical structures, these characteristics of HDR brachytherapy make it well suited for the purpose of IORT. Since many centres already own a HDR after loading machine, which can be transported to the OR for IORT, it reduces the cost of dedicated system; however, like IOERT, a shielded O.R or a shielded room in the O.R complex becomes necessary for HDR IORT. HDR IORT in most centres is delivered using surface applicators like Harrison–Anderson–Mick (HAM) applicator [8, 9] or superflab [10, 11] applicators and prescribed at 0.5–1 cm depth. These applicators are flexible, can treat relatively uneven surfaces and come in larger sizes for larger surfaces. Disadvantages of HDR IORT are reduced depth of penetration and prolonged treatment time relative to IOERT.

### KV IORT

With increasing use of IOERT in the 1980s, orthovoltage X-rays were attempted for use in IORT to reduce the shielding costs of the OR. However, poor uniformity, higher bone doses and prolonged treatment time quickly reduced the interest in their use. Recently, low-kV (20–50 kV) mobile IORT devices like Intrabeam, (Carl Zeiss AG, Germany) and Axxent Electronic Brachytherapy System (Xoft Inc., Fremont, California) are gaining popularity for use in IORT. They have steep dose gradients and do not require special shielding requirements. They come with spherical applicators and have a very limited depth of penetration of 0.5–1 cm. They are therefore best suited for spherically shaped target volumes as in breast cancer.

With a strong oncological rationale at its heart, IORT in its various forms has been tested throughout the evolution of radiotherapy and has weathered the tests of time and technology showing periodic resurgences with the advent of newer technology. The following section will focus on recently published results to describe the current role of IORT across various sites.

### Search strategy and selection criteria

A literature search was performed through the PubMed database by using the following terms: ‘intraoperative radiotherapy/IORT’, ‘head and neck cancer’, ‘breast cancer’, ‘colorectal/rectal/colon cancers’, ‘pancreas/pancreatic cancers’, ‘gastric/stomach cancer’, ‘soft-tissue sarcoma/sarcoma’, ‘paediatric/childhood cancers’, ‘gynaecological cancer’, ‘uterine/endometrial cancer’, ‘cervical/cervix cancer’, ‘renal/kidney cancer’, “bladder cancer”, and “prostate cancer”. IORT was defined as single large dose delivered intraoperatively during surgery, articles of perioperative brachytherapy with continuous low-dose rate or pulsed dose rate or HDR with multiple small fractions, delivered over subsequent days post-surgery were not included in this review. Search was limited to articles published between 1995 and 2017. Reviews, case reports and data presented, only as an abstract at conferences were excluded. Whenever updated data from the same institute was available, earlier articles with smaller numbers were not included. For the purpose of uniformity, in the respective sections, reports combining the results of primary with recurrent colorectal cancers, extremity sarcomas with retroperitoneal sarcomas and metastatic pancreatic cancers with locally advanced pancreatic cancers together were not included in the review. A total of 123 articles were finally included in the review.

## Clinical results with IORT

### Head and neck cancers

Despite the use of multidisciplinary treatment protocols locoregional recurrences occur in more than 30% of locoregionally advanced head and neck cancers [12–15]. Outcomes are poor even after surgical salvage with high rates of local failure. Re-irradiation, in this setting, has shown to improve local control [16]. However, persistent late sequelae from previous course of radiotherapy (RT) may hamper the chances of effective re-irradiation with EBRT. IORT is an attractive tool in this setting.

Many retrospective series [17–22] have demonstrated the efficacy of IORT in recurrent head and neck cancer after gross total resection (Table 1). Both IOERT and HDR IORT have been used to deliver IORT in recurrent head and neck cancer. Patients selected for IORT mainly consisted of recurrent or persistent cancers, who have been previously irradiated and delivery of sufficient doses of EBRT was not possible at the time of recurrence. Most studies have shown effective local control with acceptable complications [17, 19–22]. Resection status at salvage was the most important factor determining local control [17, 19, 21]. Microscopically residual tumours did better with IORT [23], gross residual disease however did not [20, 23]. Adjuvant EBRT after IORT appears to further improve local control, however the small sample size of these studies precludes any definite conclusions [20, 23]. Wound complications, osteoradionecrosis (ORN), fistulae, and neuropathy are the most common complications [17–22] after IOERT; however, these are rare with doses less than 20 Gy [22] and no different than that of re-irradiation with EBRT [16]. Carotid artery blow out is a rare but a fatal complication that may occur after IORT. Attempts should be made whenever possible to shield or space out the major vessels and nerves from the treatment field.

### Breast cancer

The majority of breast cancer recurrences after breast conservation surgery and whole breast irradiation (WBI) occur in the tumour bed, questioning the need for WBI. This has led to widespread adoption of accelerated partial breast irradiation (APBI) in women with early breast cancer without adverse features. IORT has seen a growing interest in early breast cancer as a modality of delivering APBI in a single fraction.

Several phase-II trials [24, 25] and prospective series [6, 26] have shown excellent early tumour control, survival, and cosmetic outcomes. Two large phase-III studies TARGIT-A (targeted intraoperative radiotherapy) [27] and ELIOT (intraoperative radiotherapy with electrons) [28], have evaluated the role of IORT as single-dose, partial breast irradiation treatment compared to standard, conventionally fractionated WBI for highly selected patients with relatively low-risk early-stage invasive breast cancer.

Table 2 summarises the relevant differences in the two trials with the 5-year results. Both the Eliot and TARGIT trials demonstrated significantly higher recurrence rates compared to WBI; however, the results were reported to be within the predefined statistical margin for equivalence/non-inferiority. Also, in both the trials, fewer skin side effects were seen in the IORT group compared to those in the WBI group.

TARGIT-A trial also reported significantly lower non-breast cancer deaths in the TARGIT group (*p* = 0.0086). This difference was attributed to fewer radiotherapy-related cardiovascular deaths in the TARGIT group; however, radiotherapy-related cardiovascular deaths may not become apparent so early in the follow-up period and these differences could have resulted due to imbalance in the treatment arms [29–32]. The TARGIT-A trial has also come in for criticism related to its statistical assumptions [33–35]. Though the trial seems to show a non-inferiority in 5-year local recurrence rates, median follow-up of all randomised patients is just 29 months which is too early to make assumptions regarding local recurrence rate at 5 years and also the authors seem to have misinterpreted the non-inferiority criterion, which require the upper confidence interval (CI) be less than the predefined non inferiority level of 2.5% [33–35].

To summarise the trials of single-dose IORT, both ELIOT trial (IOERT) and TARGIT trial (kV-IORT) demonstrated a higher recurrence rate compared to WBI, although within the equivalence margin [36]. TARGIT-A (KV-IORT) also requires a longer follow-up before drawing definite conclusions and adopting it for widespread use in place of WBI [35, 36]. It is prudent to use these techniques in a highly selected group of low-risk early-breast cancer to achieve acceptable results. Leonardi *et al* [37, 38] used the American Society for Therapeutic Radiation Oncology (ASTRO) consensus statement [39] and the Groupe Européen de Curiethérapie–European Society for Therapeutic Radiology and Oncology (GEC–ESTRO) recommendations [40] for APBI patient selection, to stratify 1822 patients treated with ELIOT outside the trial into different risk groups, 16% of women met ASTRO suitable criteria and 31% were good candidates as per GEC-ESTRO recommendations, local recurrence rates were 1.5% and 1.9%, respectively. Thirty-six per cent of women who had favourable biology disease with a luminal-A subtype also showed a very low local recurrence rate of 1.7% irrespective of the risk group. Therefore, ASTRO suitable, GEC ESTRO good and luminal-A subtype identify a subset of women, who may be safely treated with single-dose IORT with acceptable results [36–38, 41].

Though it may not be time yet for IORT to replace WBI in early-breast cancer, IORT has been investigated as a strategy for boost in limited-stage breast cancer prior to WBI. Compared to post-operative boost, IORT boost allows precise delivery to a smaller target volume separated from skin, rather than to a volume distended or distorted by seroma, thus improving accuracy and cosmesis. Also, a single-shot boost treatment significantly reduces the duration of adjuvant RT. In a pooled analysis by the International Society of Intraoperative Radiotherapy (ISIORT), IOERT has been demonstrated to be an effective boost strategy with excellent local control rates [42]. A total of 1109 unselected patients belonging to any of the risk groups were treated with IOERT boost (median 10 Gy) followed by WBI (50–54 Gy). At a median follow-up of 6 years, only 16 local recurrences were observed resulting in a local control rate of 99.2%. Grade was the only significant predictor of local recurrence, while none of the age groups demonstrated a higher recurrence rates. Efficacy of KV-IORT as a modality for intraoperative boost has been demonstrated in two large prospective series, an IORT boost of 18–20 Gy was followed by WBI, a local recurrence rate of 1.73% was observed in the study by Vaidya *et al* [43], while a 3% recurrence rate was seen in the study by Blank *et al* [44]. The TARGIT-B trial (NCT01792726), which compares EBRT boost versus an IORT boost, in patients at high risk for local recurrence who are receiving breast-conserving treatment, with standard postoperative EBRT has been launched and may provide definite answers.

IORT boost has emerged as an attractive option for boost in combination with oncoplastic surgery [45]. Oncoplastic reconstruction techniques allow for a wider resection margin while maintaining the cosmetic outcome; however, an externally delivered boost, in such cases, has higher chance of partially missing the target volume due to the tissue displacement techniques used for reconstruction. IORT allows for a precise delivery of the radiation boost directly to the tumour bed during surgery and can be followed by oncoplastic reconstruction thus maintaining the oncological safety and improving cosmetic outcome, with other added advantages like avoiding seroma formation and reducing the duration of EBRT. The Breast Centre of the University Hospital of Cologne [45, 46] has recently reported the aesthetic outcomes of X-ray IORT boost (20 Gy) combined with oncoplastic surgery in 149 patients treated since 2011, with excellent cosmetic outcomes in over 90% and seroma formation rates of 2% at 4 weeks.

### Colorectal cancers

Locally advanced rectal cancer is best managed with aggressive multimodality treatment involving chemoradiotherapy and radical resection. Most locally advanced (T3) tumours do well with this multimodality approach and local recurrences are seen in only 5–10% of patients. However, in 15% of T4 (unresectable) tumours R0 resections may not be possible [47, 48] and 10% of complete resections still develop local recurrences [47, 48] after full course chemoradiotherapy. Resection status is the most important determinant of local control and survival; incomplete resections yield few long-term survivors. There may be a case for dose escalation in locally advanced/unresectable rectal cancers with incomplete resections or at high risk of local recurrence (close margins); however, gastrointestinal tolerance limits the radiation dose delivered by EBRT. With IORT, higher doses can be delivered directly to the tumour bed without significantly increasing doses to nearby structures. This high dose may be capable of sterilising the margins even after microscopic/macroscopic residual disease.

There is increasing evidence (Table 3) to suggest that inclusion of IORT in the multi-modal treatment of locally advanced rectal cancer can lead to improved local control and survival [11, 49–51] especially in the setting of R+ resection. IORT in locally advanced rectal cancer is commonly delivered as an intraoperative boost and used in combination with pre-operative or post-operative radiotherapy with or without chemotherapy [11, 49]. Most studies have utilised IOERT and others have utilised HDR IORT for delivery of IORT in rectal cancer.

Table 3 summarises various non-randomised and randomised studies of IORT in locally advanced rectal cancers. The initial non-randomised comparisons [52–54, 57, 62] showed conflicting results with IORT after complete resection (*R*0), while some studies showed equivalent local control [54] in IORT and non IORT group^,^ others showed a significant benefit in local control with IORT [52, 57, 62]. One thing which is certain was that IORT provided significant benefit in local control and survival in patients with *R*+ resection [54, 55, 59, 61]. The only two randomised studies [56, 58] comparing the addition of IORT to standard treatment failed to show any benefit with addition of IORT in terms of local control or survival. The study by Dubois *et al*. [58] had a large proportion of T3 tumours (89%), complete resection in most patients would have likely minimised the benefits of IORT, while on the other hand, the study by Masaki *et al*. [56] was limited by small sample size and inclusion of T1/T2 patients.

Management of locally recurrent rectal (LRRC) cancers presents unique challenges. Prior irradiation in these patients limits the scope for further treatment of these patients with EBRT and is generally associated with poorer survival. IORT with its ability to limit the dose to critical structures serves as a reasonable technique for re-irradiation in LRRC. With the addition of IORT to gross total resection and EBRT, various initial series [63, 64] reported a 5-year survival of over20% even without chemotherapy.

Non-randomised studies of IORT in LRRC (Table 4) have shown a significant improvement in local control with IORT and many series have also shown a survival advantage. Recent series [65–70] have also employed Re-EBRT and chemotherapy along with IORT in patients previously treated with pelvic radiotherapy and were able to achieve survival in the range of 30–40%, using these aggressive strategies. Important factors affecting outcomes in most of these studies was completeness of surgical resection [65, 68–70] and addition of IORT boost [63, 66, 67]. EBRT during recurrent setting appears to improve the outcomes further and should be considered whenever feasible [68, 70, 71].

The complication rates in these IORT studies are variable and could range anywhere between 5% and 60%. Wound complications, gastrointestinal problems, ureteric obstruction and neuropathy are some of the frequently encountered morbidities. Wound complications were most common and in some series was quite high, upwards of 40% [50, 62, 72, 73]. Gastrointestinal fistulae and ureteric damage have an incidence ranging from 2% to 12% [50, 62, 64, 72, 73]. Plexopathy and neuropathy are late toxicities of pelvic IORT and have shown a dose-dependent relationship after IORT [50, 62, 64, 65, 72, 73]^.^

A meta-analysis [74] of studies of IORT in locally advanced and recurrent rectal cancers together, has shown a significant benefit with addition of IORT on local control, disease-free survival and overall survival. Meta-analyses of complications did not demonstrate a significant increase in urologic or gastrointestinal complications; however, a greater number of wound complications did occur [74].

### Soft-tissue sarcomas

Surgery constitutes the main treatment modality for soft-tissue sarcomas; however, surgery alone cannot provide acceptable local control rates without hampering the functionality of the limb/organ in cases of large and high grade sarcomas, thus making radiation therapy an integral component of function preserving surgery. Radiation therapy used either preoperatively or postoperatively provides acceptable local control rates after an adequate surgery with negative margins. However, in cases of advanced tumours where negative margin is not possible without mutilating surgery (retroperitoneal sarcoma) or in case of recurrent tumours, optimum doses of EBRT cannot be delivered to provide acceptable local control.

IORT has been used in such tumours to escalate doses beyond that of conventional EBRT in an attempt to improve local control rates. In extremity sarcomas, IORT has also been used to replace external boost, reducing the dose and volumes treated with EBRT, so that tolerance of normal structures like joint space, bone, and skin can be respected.

Table 5 summarises studies of IORT in extremity soft-tissue sarcomas, these studies were heterogeneous with varying proportion of recurrent tumours and incomplete resections. Use of IORT in these unfavourable patients aimed at preserving the limb while maintaining acceptable local control. IORT was mostly used in combination with function preserving surgery and moderate doses of EBRT. Recent series [82–85] of IORT demonstrate excellent LC rates and functional outcomes, comparable to the series of EBRT alone, despite including higher proportion of tumours with unfavourable factors. Dose of IORT was dependant on resection status, volume and dose of EBRT. While *R*+ disease fared equally well as *R*0 disease in the series by Call *et al* and Kretzler *et al* [79, 82], studies by Niewald *et al* and Kretzler *et al* [79, 81] reported equivalent outcomes in recurrent as well as primary disease. However, in some of the larger series, [82–85] resection status and recurrent disease were the most important factors determining local control. Limb preservation was achievable in most patients even with recurrent disease. The complications of neuropathy, contracture, and lymphedema were low, wound complications were the most common complications, and were not much different from that with EBRT [79–82].

Soft-tissue sarcomas in the retro peritoneum are difficult to remove with adequate margins due to their large size, advanced stage, and difficult location with multiple critical organs in close vicinity. Therefore, surgery is often combined with radiotherapy in order to improve the local control rate. However, the proximity of normal organs, such as viscera and neurovascular structures, has made the delivery of therapeutic doses of postoperative EBRT problematic, with higher rates of gastrointestinal complications, including disabling chronic enteritis and fistulae. These difficulties have led to adoption of IORT in the treatment regimen for retroperitoneal sarcoma since the late 1980s.

A randomised trial at the NCI [86], at a median follow-up of 8 years, showed a significantly better local control with IOERT and low-dose post-operative EBRT compared to high-dose post-operative EBRT alone (60% vs. 20%, *p* < 0.05). The IOERT arm experienced significantly more peripheral neuropathy attributed in part to use of concurrent radio-sensitisers (60% vs. 5%, *p* < 0.05), while the EBRT only arm had significantly higher GI complications. Experience from other series, summarised in Table 6, has also shown encouraging results with a favourable toxicity profile.

IORT in combination with pre-operative or post-operative RT has shown encouraging results [87–94, 97, 99]. While initial reports [87, 89, 90, 92] had higher proportion of patients receiving post-operative RT, recent series [88, 91, 93, 97–99] mostly use pre-operative RT because of the smaller volumes that are required with reduced rates of complications. Combination of pre-operative RT, gross total resection and IORT has demonstrated improved local control [91, 98] as well as survival [88, 91] compared to the non-IORT regimens in some of the recent non-randomised comparisons. Resection status and recurrent disease were the most important determinants for local control [89, 92, 94, 97]. GI toxicities, neuropathy and ureteric stenosis are the most common complications with reported rates of 10–35%. They may be dose-dependent, high single dose resulting in greater risk of complications [65, 86, 100].

Most studies of IORT shown in Tables 5 and 6 had a large proportion of recurrent tumours, emphasising the fact that IORT plays a pivotal role in the management of these locally recurrent sarcomas. In a multi-centric, long-term outcomes analysis by the Spanish Cooperative Initiative [101] for Intraoperative electron radiotherapy, 103 patients were investigated to analyse long-term outcomes of locally recurrent soft-tissue sarcoma (LR-STS) patients treated with a multidisciplinary approach. The 5-year IORT in-field control, disease-free survival (DFS), and overall survival were 73%, 43%, and 52%, respectively. Not combining EBRT with surgical resection and IOERT in patients with LR-STS was associated with a significantly increased probability of LR and IOERT in-field relapse. They concluded that low rate of severe toxic events suggests that a multimodality approach with re-resection and IOERT is feasible without prohibitive long-term side effects.

### Paediatric tumours

Most paediatric tumours are radiosensitive and radiotherapy constitutes an integral component in their management schema, more so for the unresectable and recurrent tumours, where outcomes remain dismal with chemotherapy alone. However, the use of radiotherapy, especially EBRT in the paediatric population is fraught with late effects like retarded bone and soft tissue growth, abnormal organ development and the risk of second malignancies due to the sensitive nature of these maturing tissues. Thus, there is a narrow therapeutic window within which local control and late effects, which needs to be balanced. The goal of IORT for paediatric tumours is to improve the therapeutic ratio by increasing local control while limiting these late toxicities.

Table 7 summarises various studies of IORT in paediatric tumours, though the numbers are small, IORT has been used across a wide variety of sites and histologies, as a sole radiation modality for radio-sensitive tumours like neuroblastoma [102] or in combination with EBRT for dose escalation to improve local control in sarcomas [103] or for dose de-escalation in RMS with low-dose EBRT. Oertel [103], Goodman [104] and Sole [105] *et al* included quite a number of recurrent tumours. Use of IORT in combination with surgery and EBRT provided excellent local control across most studies with acceptable toxicity.

In a study by Sole [105] *et al*, after a median follow-up of 72 months (range, 4–10 months), 10-year LC, disease-free survival, and OS was 74%, 57%, and 68%, respectively. In multivariate analysis after adjustment for other covariates, disease status (*p* = 0.04 and *p* = 0.05) and resection margin status (*p* < 0.01 and *p* = 0.04) remained significantly associated with LC and OS.

IOERT can be considered as an effective option as a part of multimodality regimen for paediatric solid malignancies, especially for patients with recurrent tumours and abdominopelvic malignancies.

### Gynaecological cancers

Recurrent gynaecological malignancies are associated with poor survival due to lack of effective salvage options. Survival rates of locally recurrent cervical cancer after prior radiation therapy are dismal. Most recurrences especially those involving the pelvic sidewall are not resectable and when resection is possible (as in central recurrences), extensive procedures like pelvic exenteration are required, which are associated with a high rate of complications and operative mortality of over 10% [108–111]. Introduction of IORT has widened the scope of patients who may be offered surgery and patients who have been previously treated with non-surgical modalities can be offered radical resection when combined with IORT. In resectable recurrences, IORT given after gross total resection can improve local control rates.

IORT has been used to treat locally advanced primary cervical cancers also; however, these series [112] are small and most of the experience comes from recurrent cancers (Table 8). IORT has shown to improve local control and thus survival in locally recurrent cancers [113–121] of the uterine cervix and endometrium, limited locoregional recurrences from endometrial cancers doing much better than recurrences from cervical cancers [119, 122–124]. The benefit of IORT is seen much more in patients with microscopic residual disease than in those with gross residual disease [113–115, 121]. Patient selection based on resection status and volume of recurrence are the most important factors determining outcome after IORT. Previously, irradiated patients when adjusted for resection status and volume of recurrence appear to fare as well as previously un-irradiated patients [115] and addition of EBRT to IORT regimen further improves the control rates [117, 119, 120, 123]. IORT does not seem to increase the rate of acute complications following surgery. Neuropathy and gastrointestinal toxicity are the most common IORT-related toxicities and occur in 5–30% of patients.

### Genitourinary cancers

#### Bladder cancer

Although multiple reports of perioperative brachytherapy in bladder cancer are available with encouraging results, there is limited data on IORT in bladder cancer, with only one small retrospective series in recurrent bladder cancer meeting our search and selection criteria. Recurrent bladder tumours after a cystectomy are associated with dismal survival rates, owing to the fact that adequate surgery is often not feasible and salvage with high doses of EBRT is difficult due to the tolerance of adjacent organs. IORT is used to deliver high doses to the tumour in an effort to improve local control. Hallemeier *et al* [128] reported the use of IOERT in 17 patients after maximal resection of disease. Pre- or post-operative EBRT was used in 94% of patients. Encouraging 2-year local control and survival was seen, completely resected tumours were associated with a significant improvement in survival compared to gross residual disease.

#### Renal cancer

Radical surgery forms the mainstay of treatment in patients with renal cell carcinoma (RCC). However, in patients with recurrent and advanced tumours, achieving complete resection with wide margins may be difficult due to proximity to the critical structures and this effects not only the local control but survival as well [129]. Adjuvant EBRT in this setting may improve local control; however, the doses achievable with EBRT is limited due to low tolerance of the surrounding structures like stomach, small bowel, contralateral kidney, liver, and spinal cord. IORT offers an attractive treatment option to escalate doses to the tumour bed, especially in cases with positive resection margins. Studies evaluating the role of IORT in the management of locally advanced and recurrent RCC are summarised in Table 9 [129–134].

Habl *et al* [132] reported outcomes with IOERT after complete surgical resection in a cohort of 17 patients with locally recurrent RCC. Although R0 resection could be achieved in only one-third of the patients, most patients failed distally, with only two local recurrences. None of the patients suffered from any acute or late radiation toxicities. One of the largest series of IOERT in RCC has been reported by Paly *et al* in a multi-institutional cohort of 98 patients. Twenty-eight per cent patients had advanced disease at presentation and 72% had recurrent disease. More than 50% had residual disease after resection. Sixty-two per cent received additional pre-operative or post-operative EBRT. An excellent local control of 72% at 5 years was demonstrated with grade 3 toxicity in 5% of patients. Higher IORT dose was associated with improved survival (*p* < 0.001). Thus, studies of IORT in RCC, though retrospective in nature demonstrate a consistently high local control rate in recurrent/advanced RCC with acceptable toxicity rates.

#### Prostate cancer

Locally advanced/high-risk prostate cancer is associated with significant risk of relapse when treated with radical prostatectomy alone, risk being the highest when the margins are positive. Adjuvant radiotherapy in this setting reduces the risk of relapse significantly [135]. IORT has been explored in high-risk prostate cancers in combination with radical prostatectomy and post-operative EBRT to improve local control via dose escalation. IORT has the added radiobiological advantage of high single dose of radiation, which improves the therapeutic gain due to low α/β of prostate. It also helps limit doses to the rectum and has been shown to have low gastrointestinal (GI) morbidity even in combination with EBRT [136]. Several small prospective series (Table 10 [136–141]) have evaluated the feasibility of this multi-modality approach in patients with non-metastatic, node-negative disease with probability of LN involvement being less than 15%. Encouraging local control and acceptable toxicity has been demonstrated even though significant proportion of patients had margin positive disease in these series [136, 139, 141]; however, long-term results are awaited.

### Upper gastro-intestinal tumours

#### Gastric cancers

Curative resection is the mainstay of treatment for gastric cancer; however, high incidence of locoregional and systemic failures, makes outcomes dismal, especially in cases with gastric serosal involvement and/or nodal involvement [142, 143]. Attempts to improve locoregional control and survival include addition of adjuvant radiotherapy/chemoradiation [144], perioperative chemotherapy [145] and extensive surgeries including D2/D3 resections [146, 147]. Despite significant improvements in disease control and survival with adjuvant chemoradiotherapy, local and regional recurrences remain high at 19% and 65%, respectively, after tri-modality therapy [144]. Therefore, there may be a case for dose escalation with IORT in advanced gastric carcinomas (especially serosal/nodal involvement) to improve local/regional control. IORT in gastric cancer involves boosting the tumour bed, remaining lymphatic networks, and nodal basins to control residual microscopic disease and improve locoregional control.

Role of IORT in gastric cancer after curative resection has been evaluated in multiple studies (retrospective, prospective, and randomised control), which have shown an improvement in locoregional control [148, 150] and survival with IORT, especially in patients with stage-II/stage-III and node-positive disease (Table 11) [1, 148, 150–154]. While initial studies of IORT involved less aggressive surgeries (D1) and infrequent use of adjuvant radiotherapy recent studies [148, 150, 153] have demonstrated a consistent benefit with IORT, even in combination with D2 resections and post-operative CTRT. Extended resections like D3 may reduce the benefit with IORT [153], however, IORT combined with a limited lymph node dissection (D1) may be associated with survival similar to extended dissection (D2/3), with lesser post-operative mortality [156]. While most studies did not show an increase in complications with the use of IORT, Drognitz *et al* [151] have demonstrated a significant increase in surgical complications with the use of IORT (44% vs. 20%, *p* < 0.05). They also did not show a benefit with addition of IORT to surgical resection. Complication rates need to be carefully weighed against improvement in locoregional control to maximise benefits with IORT [157].

#### Pancreatic cancer

Pancreatic cancer is associated with dismal survival rates even in completely resected patients. Significant proportion of patients either develop locoregional recurrence or systemic metastases. Multi-modality treatment approaches combining chemotherapy and radiotherapy in addition to surgery have resulted in some improvement in locoregional control and survival [162–164]. Attempts at radiotherapy dose escalation with EBRT have been limited due to the location of tumour. IORT can result in delivery of higher doses to the tumour bed and may improve local control and survival in resected pancreatic cancers. In unresectable tumours, IORT alone or in combination with EBRT can provide some local control along with effective palliation of symptoms.

Studies of IORT in resectable pancreatic cancers are summarised in Table 12 [165–171], though heterogeneous in proportion of *R*1 resections and use of adjuvant EBRT and/or chemotherapy, they have been consistent in showing an improvement in locoregional control [165–168]. Some studies have also shown an improvement in survival [165, 168, 172, 173]. Addition of IORT to standard treatment did not result in any increase in perioperative morbidity or late toxicity rates [165, 167–169, 173]. Stage [172, 173], *R*0 resection [166], chemotherapy [170], and pre-operative treatment [168] were other important determinants of survival in these studies. A systematic review also agreed with observations from these non-randomised studies and suggested a survival benefit with IORT in resected pancreatic patients.

Studies of IORT in unresectable pancreatic cancer (Table 12 [174–180]) on the other hand, have failed to demonstrate a survival benefit with the addition of IORT, though an improved local control was seen [174–176, 178, 181]. IORT also resulted in significant pain relief and palliation of symptoms [175, 177–179] with no additional morbidity or toxicity [175, 177, 181]. Tumour size [17, 174, 176, 180], metastasis [179], and chemotherapy [17, 174, 176] were predictors of survival in these studies of unresectable pancreatic cancer. Most of these studies included patients treated before the year 2000 and utilised post-operative radiotherapy and chemotherapy with older regimes. In the current era, pre-operative chemotherapy (± radiotherapy) with novel systemic agents (like FOLFIRINOX and nab-Paclitaxel) has shown to improve resectablity rates and survival in unresectable pancreatic cancers [182, 183]. Keane *et al* [181], evaluated the role of IORT in combination with intensive neoadjuvant chemoradiotherapy regimens and demonstrated encouraging survival rates in patients with close/positive margins and unresectable disease with no increase in toxicity. Further studies are required to better define the role of IORT in the management of pancreatic cancers, in the current era especially with the advent of novel systemic agents.

## Conclusion

Intraoperative radiation therapy is an attractive treatment option for patients with colorectal, gynaecological, intra-abdominal, head and neck, and most recently, breast cancers. IORT has been used in a multitude of roles across these sites, for dose escalation, EBRT dose de-escalation, as sole radiation modality in early-breast cancers and as a Re-irradiation modality in recurrent cancers. IORT serves its role best in combination with gross total resection and moderate doses of EBRT. Utility of IORT has been tested in the setting of a randomised control trial in early breast, retroperitoneum, gastric and colorectal cancers, the results of which support the use of IORT as a management option in these settings. However, appropriate technique and patient selection is the key to success with IORT. IORT has the potential to improve outcomes in recurrent cancers of the pelvis, head and neck and colorectum and can be considered as a supplement to gross total resection. In paediatric tumours, IORT serves to decrease late toxicities associated with EBRT. In appropriately selected patients, complication rates associated with IORT are low.

## Figures and Tables

**Table 1.** Studies of IORT in recurrent head and neck cancer after gross total resection.

Author/Year	Sample size	Study design	IORT type	IORT dose (Gy)	Prior RT (%)	Adj. RT (%)	Median follow-up	LC (%)	OS (%)	Toxicity grade 3 or >
Scala *et al* 2013 [20]	76 (100% recurrent)	Retrospective	HDR IORT	10–17.5	71	24	11	62 (2yr)	42 (2yr)	Total -6% Neuropathy-1^*^ Wound-1^*^
Ziedan *et al* 2012 [21]	96 (48% recurrent)	Retrospective (parotid cancers)	IOERT	15–20	55	57	67	68.5 (3yr)	66.1 (3yr)	Total -27% Fistula-4^*^ ORN-4^*^ Neuropathy-1^*^
Ziedan *et al* 2011 [22]	231 (89% recurrent)	Retrospective	IOERT	15–20	81	21	12	55 (3yr)	34 (3yr)	Total-27% ORN-8^*^ Neuropathy-8^*^ Fistulas-20^*^
Perry *et al* 2010 [19]	34(100% recurrent)	Retrospective	HDR IORT	10–20	100	15	23	56 (2yr)	55 (2yr)	Total-29% Wound-3^*^ ORN-1^*^ Neuropathy-1^*^
Chen *et al* 2007 [17]	137 (100% recurrent)	Retrospective	IOERT	10–18	83	26	41	61 (3yr)	36 (3yr)	Total-6% Wound-4^*^ Fistula-2^*^ Neuropathy-1^*^
Pinheiro *et al* 2002 [23]	34- SCC 10- non-SCC	Retrospective	IOERT	12.5–22.5	64	36	75.6 for living patients	46 52 (2yr)	32 50 (2yr)	Total-7% Fistula-1^*^ Neuropathy-1^*^ Carotid blowout-1^*^
Nag *et al* 1998 [18]	38 (100% recurrent)	Retrospective	IOERT	15–20	100	0	30	19 (1yr)	21% (1yr)	Total -16% Fistula-2^*^

Adj. RT: Adjuvant post-IORT RT, LC: local control, OS: overall Survival,

* Number of patients.

**Table 2.** Randomised control trials of IORT versus WBI in early-breast cancer.

Trial name	Sample size	Age	Inclusion criteria	IORT type	IORT dose	Trial design	Local recurrence (5yr)	Overall survival/mortality (5yr)
ELIOT [28]	1305 median follow-up: 5.8 years	> 48 years	any invasive cancer < 2.5 cm	IOERT	ELIOT:21 Gy/1# to tumour bed with 6–9 MeV electrons WBI:50 Gy/25# + 10 Gy/5# boost	Equivalence trial: Statistical margin was local recurrence of 7.5% in the IORT group	ELIOT:4.4% (95% C.I: 2.7–6.1) WBI: 0.4% *p* < 0.0001	ELIOT:96.8% WBI: 96.9%
TARGIT-A [27]	3451 Median follow-up: 2.5 years	> 45years	T1-2, N0, 1 IDC < 3.5 cm (If EIC or ILC on final histology, add whole breast RT)	X-ray IORT (50 kv X-ray)	TARGIT: 20 Gy to tumour bed 5–7 Gy at 1 cm depth WBI: 40–56 Gy with or without boost 10–16 Gy	Non-inferiority trial: Statistical margin was 2.5% difference in local recurrence at 5 years	TARGIT:3.3% (95% CI: 2.1–5.1) WBI:1.3% (95% CI: 0.7–2.5) *p* = 0.042	TARGIT:3.9% WBI: 5.3 *p* = 0.009

EIC: extensive intraductal component, ILC: invasive lobular cancer, WBI: whole breast RT.

**Table 3.** Studies of IORT in locally advanced colorectal cancer after gross total resection.

Author/Year	Sample size	Study design	T4 %	IORT type	IORT dose (Gy)	EBRT %	Median F/U	LC 5 year (%)	OS 5 year (%)	Toxicity grade 3 or >
Ratto *et al*. [52] 2003	43 IORT-19 No IORT-24	NRC	93	IOERT	10–15	100	74	Sx + IORT: 91 Sx − 57 *p* = 0.035	61	NR for IORT patients
Sadahiro *et al*. [53] 2004	IORT-99 No IORT-68	NRC	12	IOERT	15–25	100 (20 Gy only)	67 83	Sx + IORT-98 Sx- 84 *p* = 0.002	Sx + IORT- 79 Sx- 58 *p* = 0.002	NR for IORT patients
Ferenschild *et al*. [54] 2006	123 IORT-30 No IORT-93	NRC	25	HDR-IORT	10	100	25	R0+IORT:72 R0:71 (N.S diff) *R*^(+)^ + IORT:58 *R* ^(+)^:0 (*p* = 0.016)	R0+IORT:56 R0:66 (N.s diff) *R*+ ( + ) IORT:38 *R*+:0 (*p=* 0.026)	NR for IORT patients
Roeder *et al*. [55] 2007	243	RC	20	IOERT	10–15	86	59	R0 + IORT- 94 *R*^(+)^ + IORT-72	NR	Total-10% Proctitis-8 Fistula-7 Bowel stenosis-8
Mathis *et al*. [50] 2008	146	PC	64	IOERT	7.5–25	100	44	86	52	Total- 22% Neuropathy-3^*^ GI/GU-23^*^
Masaki *et al*. [56] 2008	44 IORT-19 No IORT-25	RCT	0	IOERT	18–20	No	34	Sx+IORT-94.7 Sx-95.5%	*p =* 0.344 N.s diff	Urinary catheter indwelling 29% vs. 3%, Rest- N.s diff
Valentini *et al*. [57] 2009	100 IORT-29 No IORT-71	NRC	100	IOERT	10–15	100	31	R0+IORT:100 R0:81 *p* = 0.014	NR	NR for IORT patients
Dubois *et al* [58] 2008	142 IORT-73 No IORT-69	RCT	100	IOERT	15–18	100	60	Sx + IORT- 91.8 Sx- 92.8 *p* = 0.6018	Sx + IORT - 69.8 Sx- 74.8 *p*=0.25	No difference in toxicity *p*=0.15
Kusters *et al* [59] 2010	605	PC Pooled analysis	29	IOERT	10–12.5	100		R0+IORT- 90.5 *R*^(+)^ + IORT-55 *p* < 0.001	67	NR
Sole *et al*. [60] 2014	335	PC	16	IOERT	10–15	100	72.6	92	75	Total-10% GI-19^*^ GU-8^*^ Neuropathy-7^*^
Holman *et al* [61] 2016	417	PC Pooled analysis	100% T4	IOERT	10–12.5	97	52	*R*0 + IORT- 87 *R*1 + IORT- 60 *R*2 + IORT- 57 *p* < 0.001	*R*0 + IORT-65 *R*1 + IORT- 34 *R*2 + IORT- 14 *p* < 0.001	NR

NRC: non randomised comparison, RCT: randomised controlled trial, PC: prospective cohort, RC: retrospective cohort, F/U: follow-up, Sx: surgery, *R*^(+)^: residual after surgery, LC:-local control, OS:-overall Survival, NR: not reported,

* Number of patients,

N.s diff: Non significant difference.

**Table 4.** studies of IORT in locally recurrent colorectal cancers.

Author/Year	Sample size	Study design	IORT type	IORT dose (Gy)	Prior EBRT %	Adj. EBRT %	Median Follow up	LC 5year (%)	OS 5year (%)	Toxicity grade 3 or >
Suzuki *et al*. [63] 1995	106 Sx+IORT:42 Sx:64	NRC	IOERT	10–30	25	98	44	Sx+IORT:60% Sx:7% at 3years	Sx+IORT:19 Sx: 7% P=0.0006	Total-36% Abcess-5 GI/GU-9 wound-3
Valentini *et al*. [66] 1999	47 Sx+IORT:11 Sx:14	NRC	IOERT	10–15	28	100	80	Sx+IORT:80 Sx:24 *p* < 0.05	Sx+IORT:41 Sx:16 N.S diff	Hydronephrosis-1 Neuropathy-0^*^
Alektiar *et al*. [75] 2000	74	RC	HDR-IORT	10–18	53	39	22	*R*0+IORT- 43 *R*^(+)^ +IORT-26 *p* = 0.02	*R*0+IORT-36 *R*^(+)^ +IORT-11 *p* = 0.04	Fistula-8^*^ Neuropathy-1^*^ Ureter-10^*^ Wound-5^*^
Lindel *et al*. [76] 2001	IORT-49 No IORT-20	NRC	IOERT	10–20	14	94	NR	*R*0 + IORT-56 *R*^(+)^ +IORT-14	*R*0 + IORT-40 *R*^(+)^+IORT-17	Wound complication-4^*^ Neuropathy-4^*^
Wiig *et al*. [67] 2002	107 Sx+IORT:59 Sx:48	NRC	IOERT	15–20	0	100	NR	Sx+IORT:50 Sx: 30 N.S diff	Sx+IORT:30 Sx: 30% N.S diff	late toxicity: NR Acute complication: N.s diff
Dresen *et al* [69]. 2008	147	RC	IOERT	10.–17.5	53	84	NR	*R*0 + IORT-69 *R*1 + IORT-29 *R*2 + IORT-28 *p* < 0.001(3yr)	*R*0 + IORT-59 *R*1 + IORT-27 *R*2 + IORT-24 *p* < 0.001(3yr)	Neuropathy-16^*^ Ureter stenosis-4^*^
Haddock *et al*. [65] 2011	607	PC	IOERT	7.5–30	45	96	44	*R*0 + IORT-79 *R*1 + IORT-56 *R*2 + IORT-49 *p* < 0.001	*R*0 + IORT-46 *R*1 + IORT-27 *R*2 + IORT-16 *p*< 0.001	Total-11% Wound-42^*^ Neropathy-18^*^
Roeder *et al*.[70] 2012	97	PC	IOERT	10–20	44	52	33	*R*0 + IORT-82 *R*1 + IORT-41 *R*2 + IORT-18 *p* < 0.001(3yr)	*R*0 + IORT-80 *R*1 + IORT-37 *R*2 + IORT-35 *p* < 0.001(3yr)	Acute: Abscess /fistula-16 Late: Neuropathy-8^*^ Ureter stenosis-3^*^
Calvo *et al*. [68] 2013	60	RC	IOERT	10–15	50	47	36	44 *R*0 vs *R*1: HR-2.09, *p* = 0.05	43 *R*0 vs R1:HR-2.9, *p* = 0.05	Total:42% Fistula-4^*^ Neuropathy-4^*^ GI-4^*^
Holman *et al*. [71] 2017	565	PC pooled analysis	IOERT	10–20	46	95	40 months In survivors	*R*0 + IORT-72 *R*1 + IORT-36 *R*2 + IORT-39 *p* < 0.0001	*R*0 + IORT-48 *R*1 + IORT-25 *R*2 + IORT-17 *p* < 0.0001	

NRC: non randomised comparison, RCT: randomised controlled trial, PC: prospective cohort, RC: retrospective cohort, Sx: surgery, *R*^(+)^: residual after surgery, *R*0: no residual after surgery, LC: local control, OS: overall survival, NR: not reported,

* Number of patients,

N.s diff: non-significant difference.

**Table 5.** Studies of IORT in extremity soft tissue sarcoma in combination with function preserving surgery and moderate doses of EBRT (40-50Gy).

Author/Year	Sample size	Study design	IORT type	IORT dose Gy	EBRT %	Median follow-up	LC 5 year	DFS 5 year	OS 5 year	Complications
Edmonson *et al*. [77] 2001	39 (Recurrent-3%, *R*+:38%)	Retrospective	IOERT/HDR IORT	10–20	100	70	90^α^	NR	80	NR for IORT
Azinovic *et al*. [78] 2003	45 (Recurrent-42%, *R*+:33%)	Retrospective	IOERT	10–20	80	93	80 *R*0 vs *R*1: 88 vs 57 *p* = 0.04	NR	64	Wound complication -4^*^ Neuropathy-5^*^ Fracture-2^*^
Kretzler *et al*. [79] 2004	28 (Recurrent -57%, *R*+:39%)	Retrospective	HDR/IOERT	12–15	90	55	84	54	66	Total-24% Neuropathy-1^*^ Fractures-2^*^ Contracture-2^*^
Oertel *et al*. [80] 2006	153 (Recurrent - 38%, *R*+: 30%)	Retrospective	IOERT	10–20	100	33	78	NR	77	Wound-17% Neuropathy-7^*^ Lymphededma-6^*^
Niewald *et al*. [81] 2009	38 (Recurrent -24%,)	Retrospective	HDR-IORT	8–15	100	27	63	NR	57	Skin-42% Neuropathy-0
Call *et al*. [82] 2012 ¥	61 (Recurrent -21%, *R*+:18%)	Retrospective	IOERT	7.5–20	100	70	91	80	72	Wound-3.2% Neuropathy-1^*^
Calvo *et al*. [83] 2014	159 (*R*1-16%)	Retrospective pooled	IOERT	10–20	100	53	82 *R*0 vs *R*1: *p* = 0.009	62	72	Acute skin/wound- 16% Neuropathy-6^*^ Lympedema-7^*^
Roeder *et al*. [84] 2014	34 (*R*1-12%)	Prospective	IOERT	10–15	100	43	97	66	79	Neuropathy-1^*^ osteonecrosis-1^*^ Joint dysfunction-1^*^
Roeder *et al*. [85] 2016	183 (Recurrent -22%, *R*1 - 32%)	Retrospective	IOERT	8–20	100	64	86 *R*0 vs *R*1: 92 vs 75 *p* = 0.019	61	77	Total-19% Wound-15^*^ Neuropathy-14^*^ osteonecrosis-11^*^

*R*+: Residual after surgery, *R*0: no residual after surgery, LC: local control, OS: overall Survival, DFS: disease-free survival, NR: not reported,

^*^ Number of patients,

¥ call *et al* included only upper extremity tumours,

α -crude rate

**Table 6.** Studies of IORT in Retroperitoneal sarcoma.

Author/Year	Sample size	Study design	IORT type	IORT dose (Gy)	EBRT %	Median follow-up	LC 5 year (%)	OS 5 year (%)	Toxicity grade 3 or >
Sindelar *et al*. [86] 1993	35 GTR-100%	RCT Sx + IORT + low-dose PORT Vs Sx + high-dose PORT	IOERT	20	100	96	IORT + PORT- 60%’ PORT-20%	-	Neuropathy IORT + PORT-60% PORT-5% Enteritis IORT + PORT-13% PORT-50%
Alektiar *et al*. [87] 2000	32 Recurrent -62%, GTR-94%	Retrospective cohort	HDR-IORT	12–15	78	33	62	45	GI -18% Neuropathy-0%
Gieschen *et al*. [88] 2001	37 IORT-20 No IORT-17 Recurrent-22% GTR-78%	Retrospective cohort	IOERT	10–20	100 100	38	IORT-83 No IORT-61 p = 0.197¥	IORT-74 No IORT-30 p = 0.044¥	Total-20% Neuropathy-1^*^ Fistula-2^*^
Peterson *et al* [89] 2002	87 Recurrent-50%, GTR-84%	Retrospective cohort	IOERT	8.75–30	89	42	59	47	GI-14% Neuropathy- 10%
Bobin *et al*. [90] 2003	24 Recurrent-79% GTR-92	Retrospective cohort	IOERT	8–22	92	53	46	56	Total-8% Neuropathy-2^*^
Pierie *et al*. [91] 2006	IORT-14 No IORT-27 Recurrent-0% GTR-100%	Retrospective cohort	IOERT	10–20	100 100	27	NR	IORT-77 No IORT-45 *p* = 0.38	GI-1% Neuropathy-3%
Krempien *et al*. [92] 2006	67 Recurrent-61%, GTR-82%	Retrospective cohort	IOERT	12–20	67	30	40	64 *R*0:87 *R*+:50 *p* < 0.01	Fistula-3^*^ Neuropathy-5^*^ Urethral stenosis-2^*^
Pawlik *et al*. [93] 2006	72 IORT-22 No IORT-50 Recurrent-25%, GTR-75%	Prospective cohort	IOERT	15	100	40	60¥	50	NR
Ballo *et al*. [94] 2007	83 IORT-18 No IORT-63 Recurrent-28%, *R*+-47%	Retrospective cohort	IOERT	10–15	100	47	IORT-46 No IORT-51 *p* = 0.9	NR	NR
Dziewirski *et al*. [95] 2010	57 Recurrent -74%, GTR-85%	Prospective cohort	HDR-IORT	20	60	20	51	55	NR
Sweeting *et al*. [96] 2013	18 Recurrent-28%, GTR-100%	Retrospective cohort	IOERT	10–20	94	43	64	72	NR
Roeder *et al*. [97] 2014	27 Recurrent-15%, GTR-100%	Prospective cohort	IOERT	10–20	100	33	72	74	Total-6% late toxicity
Stucky *et al*. [98] 2014	63 IORT-37 Sx only-26 Recurrent-36%,GTR-89%	Retrospective cohort	IOERT	10–20	100 0	45	IORT-89 Sx-46 *p* = 0.03	IORT-60 No IORT-60	Ureteral stricture-1 No grade-3 neuropathy
Gronchi *et al*. [99] 2014	83 IORT-14pts only Recurrent-24% GTR-84%	Prospective cohort	IOERT	10–12	88	58	63	59	NR

RCT: randomised control trial, PORT: post-operative RT, Sx: surgery, LC: local control, OS: overall survival, DFS: disease-free survival, NR: not reported,

* Number of patients

^¥^ In GTR patients,

α-crude rate

**Table 7.** Studies of IORT in various paediatric tumours.

Author/Year	Sample size	Study design	IORT type	IORT dose (Gy)	EBRT %	Median Follow-up	LC 5 year (%)	OS 5 year (%)	Toxicity grade 3 or >
Haase *et al* [102] 1994	25 (neuroblastoma)	Prospective, single arm	IOERT	10–17	NR	51 (mean)	75	63	No late effects at 5-year follow-up
Nag *et al* [106] 2003	13 (5 metastatic)	Retrospective	IOERT	10–15	38	42	72 (3yr)	31 (3yr)	late morbidity-30%
Goodman *et al* [104] 2003	66 (35% recurrent)	Retrospective	HDR-IORT	12–15	44	12	56 (2yr)	54 (2yr)	Late morbidity-12%
Oertel *et al* [103] 2005	18 (17% recurrent)	Retrospective	IOERT	8–15	100	54.5	95 (3yr)	83 (3yr)	late morbidity-33% Loss of Limb-1^*^ Neuropathy-1^*^
Stauder *et al* [107] 2011	20	Retrospective	IOERT	7.5–25	100	139	77 (10yr)	65 (10yr)	No grade 3 or more late effects or second primary
Sole *et al* [105] 2015	71 (35% recurrent)	Retrospective	IOERT	7.5–20	100	72	68 (10yr)	74 (10yr)	Late morbidity-13% Neuropathy-4^*^ Necrosis-2^*^ Lymphedema-2^*^

LC: local control, OS: overall survival, DFS: disease-free survival, NR: not reported,

^*^ Number of patients.

**Table 8.** Studies of IORT in recurrent gynaecological malignancies after gross total resection.

Author/Year	Sample size	Site	Primary/Recurrent	IORT type	IORT dose (Gy)	Prior EBRT %	Present EBRT %	Median follow-up	LC 5yr (%)	OS 5yr (%)	Toxicity grade 3 or >
Haddock *et al*. [113, 115] 1996	63	Cervix-40 Endometrium-16 Others-8	Primary-16% Recurrent-84%	IOERT	8–25	0 70	100 63	NR	61	27	Total-17% GI-5^*^ Neuropathy-2^*^
Mahe *et al*. [124, 125] 1996	70	Cervix	Recurrent-100%	IOERT X ray IORT	10–30	97	42	15	21 (3yr)	8 (3yr)	Total-14%Neuropathy-5^*^ Ureteral obstruction-4^*^
Del carmen *et al*. [126] 2000	15	Cervix-5Endometrium-3 Vagina-7	Recurrent-93%	IOERT	10–22.5	60	6	NR	NR	74	Neuropathy-4^*^ GU-3^*^ Lymphedema-2^*^
Martinez-monge *et al*.[116] 2001	67	Cervix	Primary-46% Recurrent-54%	IOERT	10–25 10–20	86	97 14	58 19	80.5 49	58 (10yr) 14 (10yr)	Total-14.9% Neuropathy-1^*^ Chronic pain-8^*^
Gemignani *et al*. [114] 2001	17	Cervix-9 Endometrium-7 Vagina-1	Recurrent- 100%	HDR-IORT	12–15	82	12	20	67 (3yr)	54 (3yr)	GI-4^*^ Neuropathy-3^*^ Wound-4^*^
Dowdy *et al*. [126] 2006	25	Endometrium	Recurrent- 100%	IOERT	10–25	56	84	34	84	*R*0-71 *R*1-47 *R*2-0	Neuropathy-8^*^ GU-5^*^ Fitula-5^*^
Tran *et al*. [118] 2007	36	Cervix-17 Endometrium-11 Others-8	Recurrent-89%	X-ray IORT	6–17.5	72	53	50	44	47	Total-27% Wound-4^*^ GI-1^*^
Barney *et al* [119].2013	86	Cervix-100	Recurrent-85%	IOERT	6–25	81	71	32	Primary-70 Recurrent-61% (3yr)	25 (3yr)	GI-4^*^ GU-4^*^ Neuropathy-1^*^
Calvo *et al*.[120] 2013	35	Cervix-20 Endometrium-7 Others-8	Recurrent- 100%	IOERT	10–15	71	46	46	58	42	Fistula-5^*^ Ureter stenosi-2^*^ Neuropathy-1^*^
Backes *et al*.[127] 2014	32	Cervix-21 Others-11 IORT in 66% only	Recurrent- 100%	IOERT/HDR-IORT	10–20	100	0	NR	PE+IORT-10Mon LEER+IORT-9Mon PE-33Mon	PE+IORT-10Mon LEER+IORT-10Mon PE-41Mon	NR
Foley *et al*.[121] 2014	32	Cervix-21 Endometrium-6 Others-5	Rccurent-81%	IOERT	10–22.5	88	20	26	*R*1-73 *R*2-71	*R*1-77 *R*2-55 *p* = 0.001	Total-47% GU-2 Lymphedema-2
Sole *et al*. [117] 2014	61	Cervix-18 Endometrium-32 Ovarian-9 Vagina-2	Recurrent Pelvic-57% Para-aortic- 43%	IOERT	10–15	66	48	42	65	45	Total-20% Fistula-5^*^ Neuropathy-1^*^ Wound-3^*^
Arians *et al*.[122] 2016	36	Cervix-18 Endometrium-12 Vulva-6	Recurrent- 100%	IOERT	10–18	76 25 100	11 17 17	14	Cervix-0 Endometrium-40 Vulva-20 *p* = 0.017	Cervix-6 Endometrium-50 Vulva-17 *P* = 0.038	Wound complications-5^*^ Neuropathy-4^*^ Lymphedema-3^*^

Sx: surgery, LC: local control, OS: overall survival, NR: not reported,

^*^ Number of patients

PE: pelvic exenteration, LEER: laterally extended endopelvic resection, Mon: median survival in months.

**Table 9.** Studies of IORT in bladder and renal cancers.

Author/Year	Sample size	Study design	IORT type	IORT dose (Gy)	Prior EBRT %	Present EBRT %	Median follow-up months	LC 5 year %	OS 5 year %	Toxicity grade 3 or >
Hallemeier *et al*. [128] 2013	Bladder-13, Ureter-4 Recurrent-88%	Retrospective cohort	IOERT	10–20	24	94	43.2 In survivors	51 (2 yr)	16 (5 yr) *R*0+*R*1: 56 *R*2-11 *p* = 0.03 (2 yr)	Total-12% Ureter stricture-4 Fistula-1
Paly *et al*. [134] 2014	98-RCC Recurrent-72%	Retrospective	IOERT	9.5–20	–	63	42 In survivors	76	Advanced-37 Recurrent-55	Total-5% Pancreatic leak-3^*^ gastritis-1^*^ ARDS-1^*^
Calvo *et al*. [130] 2013	25-RCC Recurrent-40%	Retrospective	IOERT	9–15	-	60	266	80	38	Total-24%
Habl *et al*. [132] 2013	17-RCC Recurrent-100%	Retrospective	IOERT	10–20	–	65	18	91 (2 yr)	73 (2 yr)	None
Hallemeier *et al*. [129] 2012	22-RCC Recurrent-86%	Retrospective	IOERT	10–20	-	95	119 In survivors	73	40 *R*0-80 *R*1-29 *p* = 0.057	Total-23% ARDS-1^*^ Pancreatic pseudocyst-1^*^ Perforated ulcer-2^*^
Master *et al*. [133] 2005	14-RCC Recurrent-100%	Retrospective	IOERT	12–20	–	NR	66 (mean in survivors)	85 (crude rate)	30	NR
Eble *et al*. [131] 1998	11-RCC Recurrent-73%	Retrospective	IOERT	15–20	-	100	24	100	47 (4 yr)	Wound-2^*^ None IORT related

RCT: randomised control trial, Sx: surgery, LC: local control, OS: overall survival, DFS: disease-free survival, NR:-not reported,

^*^ Number of patients

ARDS: acute respiratory distress syndrome.

**Table 10.** IORT studies for prostate cancers.

Author/Year	Sample size	Study design	IORT type	IORT dose (Gy)	Present EBRT %	Median follow-up months	LC 5 year %	OS 5 year %	Toxicity grade 3 or >
Krengli *et al*. [139] 2010	38 (intermediate– high-risk Pca, Margin +ve: 71%)	Prospective	IOERT	10–12	Margin +ve and/or ECE	18.2	NR	NR	No grade 3 complications
Rocco *et al*. [136] 2009	IORT-33 (intermediate– high-risk Pca, Margin +ve: 24%) RP-100	Matched pair analysis	IOERT	12	IORT-88 *R*1, pT4, N+ RP-44	16	IORT-97 RP-86 (bRFS)	100 ( 2yr)	Acute grade ≥ 2 IORT vs. RP GU-7% vs. 5% GI-3% vs. 4% Lategrade ≥ 2 IORT vs. RP GU-3% vs. 1% GI-0% vs. 1%
Saracino *et al* [141] 2007	34 (intermediate-risk Pca, Margin +ve: 41%)	Prospective	IOERT	16–22	None	41	77.3 (3-yr bRFS)	71 (3-yr)	None
Orrechia *et al*. [140] 2007	11 (high-risk Pca)	Prospective	IOERT	12	67	NR	NR	NR	1 had acute symptomatic lymphocele
Kato *et al*. [138] 1998	54 Stage B2-D1^α^ No RP	Prospective	IOERT	25–30	100 (30 Gy)	54	83 (LC) 75 (bRFS)	NR	Late rectal toxicity-7% (No toxicity with IORT of 25 Gy)
Higashi *et al*. [137] 1998	35 Stage B-C^α^ No RP	Prospective	IOERT	25–30	100 (30 Gy)	NR	NR	Stage B-92 Stage C-87	NR

RCT: randomised control trial, Sx: surgery, LC: local control, OS: overall survival, DFS: disease-free survival, NR: not reported, *Number of patients, RP: radical prostatectomy, Pca: prostate cancer, ECE: extra capsular extension, *R*1: margin +ve, *N*+: node-positive disease, bRFS: biochemical relapse-free survival,

^α^ Whitmore-Jewett staging system [Whitmore 1956, Jewett 1975].

**Table 11.** IORT studies for gastric cancers.

Author/Year	Sample size	Study design	IORT type	IORT dose (Gy)	EBRT (%)	Type of nodal dissection	Median follow-up (months)	LRC	OS 5 year (%)	Toxicity grade 3 or >
Sindelar *et al*. [149] 1993	Sx+IORT-27 Sx+/-EBRT-33	RCT	IOERT	20	0 72	NR	84	37 8 *p* < 0.001	25 months (M.S) 21 months (M.S) *p* = 0.99	Fistula: IORT-4 vs. Sx-5^*^ Enteritis: IORT-0 vs. Sx-2^*^
Abe *et al* [1] 1995	IORT-94 No IORT-127	NRC	IOERT	28–35	none	NR	NR	NR	Stage II: IORT-78 No IORT-66 Stage III: IORT-60 No IORT-51 Stage IV: IORT-33 No IORT-14 (all N.S)	NR
Avizonis *et al*.[158] 1995	27	Prospective phase II	IOERT	12.5–16.5	79	NR	NR	85	47 (2yr)	Acute toxicity-14% Late-7%
Ogata *et al*. [152] 1995	IORT-58 No IORT-120	Retrospective	IOERT	12	None	D2	NR	NR	Stage II: IORT-100, No IORT-63 (4yr) Stage III: IORT-55 No IORT-35 (8yr) Stage IV: IORT-12 No IORT-13 (5yr)	None
Coquard *et al*. [156] 1997	63 (*R*0-92%)	Retrospective	IOERT	12–23	48	D1-89%	61 in survivors	76 (crude rate)	47	None attributed to IORT
Skoropad *et al*. [154] 2000	78 Pre-op RT+IORT+ Sx vs. Sx alone	RCT	IOERT	20	100 (Pre-op RT-20 Gy/5^#^)	D1	NR	NR	Entire cohort: IORT:21months No IORT: 9months (*P* = 0.311) Node +ve and Advanced stage: IORT vs. No IORT: *p* < 0.05	Similar acute toxicity in both arms Higher pancreatitis surgery alone. No RT late toxicity
Weese *et al*. [159] 2000	16 (IORT-56%)	Prospective	IOERT	10	88	D2	27	93 (crude rate)	66 (crude rate at 3yr)	NR
Glehen *et al*. [160] 2003	42 (All Node +ve, *R*0-93%)	Retrospective	IOERT	12–15	97	NR	131	79	45	NR
Miller *et al*. [161] 2006	50 (*R*0-42%, recurrent-26%, oesophagus-14%)	Retrospective	IOERT	10–25	96	NR	19	75	15 (5 yr)	Acute-48% Chronic-26% GI-6^*^ Overall treatment-related mortality-6%
Qin *et al*. [153] 2006	IORT-106 No IORT-441	NRC	IOERT	10–30	None	D2/3	NR	NR	Stage III D2: IORT- 60% No IORT-36% *p* < 0.005 Stage III D3: IORT-61% No IORT-56% *p* > 0.05	NR
Drognitz *et al*. [151] 2007	IORT-61 No IORT-61 (*R*0-100%)	NRC	IOERT	15–25	None	D2	56	90	IORT-58% No IORT-59% *p* = 0.99	Perioperative mortality-4.9% both groups Surgical morbidity: IORT:44% No IORT: 20%
Fu *et al*. [148] 2008	97 IORT-46 No IORT-51 (*R*0-90%)	Prospective	IOERT	12–15	100% CTRT	D2	24	77 IORT- 77 No IORT- 63 *p* = 0.05, (3Yr)	44 IORT-56 No IORT-47 *p* = 0.20, (3Yr)	Late toxicity-3^*^ No difference among groups
Zhang *et al*. [150] 2012	97 IORT-46 No IORT-51 (*R*0-90%)	Prospective	IOERT	12–15	100% CTRT	D2	37	50 IORT-50 No IORT-35 *p* = 0.04	26 IORT-28 No IORT-26 *p* = 0.4	IORT vs. No IORT Acute-39% vs. 37% Late-.10% vs. 0%, *p* = 0.02 Enteritis-1^*^ Haemorrhage-4^*^
Calvo *et al*. [155] 2012	32 (*R*0-100%)	Retrospective	IOERT	10–15	47	D2	40	84	55	Acute GI-5^*^

RCT: randomised control trial, NRC: non-randomised comparison, Sx: surgery, LC: local control, OS: overall survival, DFS: disease-free survival, NR: not reported,

^*^ Number of patients,

^#^ fractions,

Pre-op: pre-operative, M.S: median survival, N.S: non-significant.

**Table 12.** Studies of IORT in the management of pancreatic cancers.

Author/Year	Sample size	Study design	IORT type	IORT dose (Gy)	EBRT %	Median follow-up	LC 5 year (%)	OS 5 year (%)	Toxicity grade 3 or >
Mohiuddin *et al*. [178] 1995	49 UR-PC (Resected-0%)	Retrospective	IOERT	10–20	100 (Post-op)	28	69	7 (4yr)	Acute toxicity-14% GI bleeding-2^*^ Late toxicity-19% GI bleeding-3^*^ Obstruction-2^*^
Nishimura *et al*. [179] 1997	Resected-157 IORT- 55 No IORT-102 Unresectable-175 IORT-71 No IORT-104	Retrospective	IOERT	12–33 Gy	Resected- 70 Unresectable-87 (Pre-op or post-op)	NR	NR	Resected: IORT-16 No IORT-0 (2yr) Unresectable: IORT-14 No IORT-0 *p* < 0.05 (2yr)	IORT: Late toxicity: gastric ulcer-18^*^ Intestinal perforation-4^*^
Ma *et al*. [177] 2004	81 UR-PC (Resected-0%) IORT-18 IORT+EBRT-25 EBRT-16 Palliative Sx-22	Retrospective	IOERT	15–25	80 (Post-op)	NR	NR (60% complete pain relief with IORT)	10.7 (M.S) 12.2 (M.S) 5.1 (M.S) 7 (M.S)	IORT vs IORT+EBRT: Delayed gastric emptying: 3 vs 2^*^
Willet *et al*. [180] 2005	150 UR-PC (Resected-0%)	Retrospective	IOERT	15–20 Gy	100 (Pre/post-op RT)	NR	NR	7 (3Yr)	Post-operative complications- 20% Late toxicity- 15% Upper GI bleed-16^*^
Jingu *et al*. [176] 2012	322 Resected-83 Unresectable- 109 Metastatic-130 (exclude)	Retrospective	IOERT	20–30	29 (post-op RT)	38	64	9 *R*2:HR-2.03, *p* < 0.001	Late toxicity GI-4^*^
Cai *et al*. [174] 2013	194 UR-PC (Resected-0%)	Retrospective	IOERT	10–25	97% (Pre-op CTRT)	12	38 (3yr)	6 (3yr)	Acute toxicity- 21% Late toxicity-14% Haemorrhage-23^*^
Chen *et al*. [175] 2016	247 UR-PC (Resected-0%)	Retrospective	IOERT	10–20	51% (Post-op CTRT)	10	35 (3yr) Complete pain relief-70%	7.2 (3yr)	Post-operative complications-14% Fistula-11^*^ Haemorrhage- 7^*^
Keane *et al*. [181] 2016	68 UR-PC After NACTRT Resected-41 IORT-22 (R1-73%) No IORT-19 Unresectable-18 (IORT-17)	Retrospective	IOERT	8–13 15–17	100 % (NACTRT)	21	NR	35.1 (M.S) 24.5 (M.S) 24.8 (M.S)	No significant difference in post-operative complications
Kokubo *et al*. [172] 2000	138 R/BR-PC (Resected-100%, *R*1-29%)	Retrospective	IOERT	20–30	45 (Pre-op:13% Post op-47% Both-40%)	NR	NR	*R*0:19 *R*1:4 *p* < 0.005 (2-yr cause specific survival)	Acute toxicity- none Late toxicity- GI ulcers-20% Perforation-2^*^
Alfieri *et al*. [165] 2001	46 R/BR-PC (Resected-100%) IORT-26 (R1-10%) No IORT-20 (R1-13%)	NRC	IOERT	10	100 0 (Post-op)	82	IORT-58 No IORT-30 *p* < 0.001	IORT-16 No IORT-6 *p* = 0.06	IORT vs. No IORT Acute morbidity- 57% vs. 43% (*p* = 0.1) Perioperative mortality- 8% vs. 9%
Reni *et al*. [173] 2001	127 R/BR-PC (Resected-82%) IORT- 127 (*R*0-1:104, *R*2-23) No IORT- 76 (*R*0-1:62, *R*2-14)	Retrospective	IOERT	10–25	32 20 (Post-op)	21 (in survivors)	Stage I-II: IORT-73 No IORT-40 Stage III-IVA: IORT-50 No IORT-45	Stage I–II: IORT-22 No IORT-6 Stage III-IVA: IORT-3 No IORT-5	IORT vs. No IORT: Acute toxicity: N.S difference Chronic toxicity: Abdominal pain 15 vs. 22% Late GI bleed-6 vs. 3% Stenosis-3% vs. 0%
Messick *et al*. [169] 2008	49 R/BR-PC (Resected-100%, R1-74%) IORT-22 No IORT-27	NRC	IOERT	10–12	76 64	10.1 13.3	IORT-82 No IORT-88 (N.S)	IORT-20 (M.S) No IORT-13 (M.S) (N.S)	IORT vs. No IORT: Delayed gastric emptying-6.7 vs. 4.2% Wound infection-4.5 vs. 22% Pancreatic fistula 10 vs. 4.8% (N.S)
Valentini *et al*. [171] 2008	26 R/BR-PC Resected-100%, *R*1-4%	Retrospective	IOERT	10	100 (Post-op: 65%)	102 in survivors	57	15	Perioperative complications-11%
Showalter *et al*. [167] 2009	R/BR-PC IORT-37 (*R*1-2:43%) No IORT-46 (*R*1-2: 30%)	Retrospective	IOERT	10–20	74 66 (Post-op RT)	NR	IORT-79 No IORT-61 *p* = 0.19	IORT-21 (M.S) No IORT-19 (M.S)	Perioperative complications-46% vs. 40% (N.S)
Valentini *et al*. [168] 2009	270 Resected-81% (*R*1-27%)	ISIORT Pooled analysis	IOERT	7.5–25	64 (pre-op:24 Post-op:40)	96	23 Pre-op RT vs. post-op RT vs. IORT alone *p* < 0.0001	18 IORT + Pre-op RT vs. IORT + post-op RT vs. IORT alone *p* < 0.0001	Acute toxicity- None > G2 Late-NS
Ogawa *et al*. [170] 2010	210 R/BR-PC Resected-100%, R1-32%	Retrospective	IOERT	20–30	30	26	84 (2yr)	42 (2yr)	Late toxicity GI-7^*^
Calvo *et al*. [166] 2013	60 R/BR-PC (Resected-83%, *R*1-43%) IORT-29 No IORT-31	Prospective	IOERT	10–15	100% (Pre-op CTRT-32%)	16	58 No IORT: HR-6.75, *p* = 0.01	20	Perioperative complications-43% (N.S) Chronic-17% Neuropathy-4^*^ GI-3^*^

RCT: randomised control trial, NRC: non-randomised comparison, Sx: surgery, LC: local control, OS: overall survival, DFS: disease-free survival, NR: not reported,

^*^ Number of patients,

^#^ fractions, Pre-op: pre-operative, Post-op: post-operative, M.S: median survival in months, N.S: non-significant, R/BR-PC: resectable/borderline resectable pancreatic cancer, UR-PC: unresectable pancreatic cancer, NACTRT: neoadjuvant chemoradiotherapy, Resected: complete resections (R0/R1), Unresectable: R2/palliative resections.
